# Sexually Dimorphic Outcomes after Neonatal Stroke and Hypoxia-Ischemia

**DOI:** 10.3390/ijms19010061

**Published:** 2017-12-26

**Authors:** Christiane Charriaut-Marlangue, Valérie C. Besson, Olivier Baud

**Affiliations:** 1 U1141 PROTECT, Inserm, Université Paris Diderot, Sorbonne Paris Cité, Hôpital Robert Debré, 48 boulevard Sérurier, 75019 Paris, France; valerie.besson@parisdescartes.fr (V.C.B.); olivierfrancois.baud@hcuge.ch (O.B.); 2 EA4475—Pharmacologie de la Circulation Cérébrale, Faculté de Pharmacie de Paris, Université Paris Descartes, Sorbonne Paris Cité, 4 Avenue de l’Observatoire, 75006 Paris, France; 3 Division of Neonatology and Pediatric Intensive Care, Children’s University Hospital of Geneva and University of Geneva, 1205 Geneva, Switzerland

**Keywords:** stroke, hypoxic-ischemic encephalopathy, microglia, gender, developing brain, oxidative stress, cell death

## Abstract

Cohort studies have demonstrated a higher vulnerability in males towards ischemic and/or hypoxic-ischemic injury in infants born near- or full-term. Male sex was also associated with limited brain repair following neonatal stroke and hypoxia-ischemia, leading to increased incidence of long-term cognitive deficits compared to females with similar brain injury. As a result, the design of pre-clinical experiments considering sex as an important variable was supported and investigated because neuroprotective strategies to reduce brain injury demonstrated sexual dimorphism. While the mechanisms underlining these differences between boys and girls remain unclear, several biological processes are recognized to play a key role in long-term neurodevelopmental outcomes: gonadal hormones across developmental stages, vulnerability to oxidative stress, modulation of cell death, and regulation of microglial activation. This review summarizes the current evidence for sex differences in neonatal hypoxic-ischemic and/or ischemic brain injury, considering the major pathways known to be involved in cognitive and behavioral deficits associated with damages of the developing brain.

## 1. Introduction

Hypoxic-ischemic (HI) encephalopathy (HIE) and stroke are two major causes of long-term neurological sequelae and cerebral palsy in children [1,2,3]. Groups at risk for ischemic stroke are newborns (the first 28 days of life), especially full-term infants, and older children with sickle cell anemia, or congenital heart defects [4]. HIE and stroke result in a spectrum of developmental disturbances including epilepsy, mental retardation, visual and hearing problems, cognitive disorders, and other neurophysiologic handicaps leading to behavioral disorders associated with cerebral palsy [5,6]. Male sex is recognized to be a risk factor for neonatal HIE during perinatal period [7]. Males are two times more likely to experience prenatal anoxia, hemorrhage, and infection and ischemic injury appears to be more common in boys regardless of lesion types [8,9]. Similarly, in a pre-clinical experiment and once an injury occurs, male sex was also associated with more limited recovery and plasticity, leading to increased incidence of long-term cognitive deficits compared to females with similar HI injury [10]. Sex differences in inflammatory responses, microglial activation, metabolic profile, brain structure and plasticity have been suggested to have a role [11,12].

**Figure 1:**
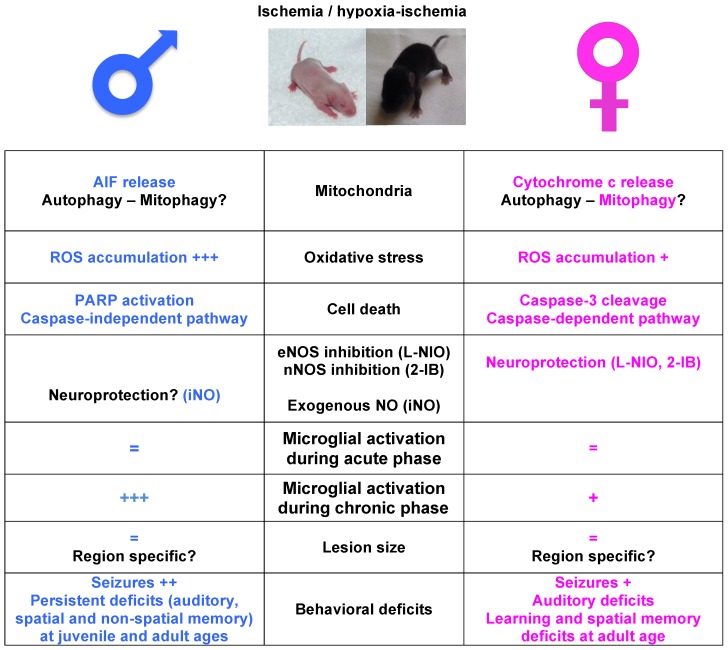
Schematic illustration of sexual dimorphism pathways after neonatal ischemia and hypoxia-ischemia. Major molecular pathways, including the lesion size and behavioral deficits (**middle** column), in male (**blue**, **left** column) and female (**pink**, **right** column), with relative agreements in the literature. In black, lack of consensus in the literature and/or single report. AIF: apoptosis inducing factor; eNOS: endothelial nitric oxide synthase; nNOS: neuronal NOS; iNO: inhaled NO; ROS: reactive oxygen species.

This review summarizes the biological basis for sex differences observed in neonatal hypoxic-ischemic and/or ischemic brain injury. Cellular mechanisms of acute injury will be discussed regarding cell death and inflammatory pathways coupled with differences in cognitive and behavioral deficits that may contribute to these clinical differences (for summarize see Figure 1).

## 2. Preclinical Models of Brain Injury and Sexual Dimorphism

Studies regarding neurodevelopmental parallels across species have provided the basis for the use of relevant animal models to study injury of the developing brain [13,14]. Using these data, there is now compelling evidence that P3 and P5 rat or mouse pups are suitable for studies of preterm injuries, whereas models using P7 to P10 rat or mouse can be more representative of injuries in near-term and full-term babies [15]. Several appropriate models to test promising therapies against neonatal HI and/or stroke have been introduced. These models have been designed in the rat and mouse brain and most of them are impeded by the great variability observed in the lesion size and brain area damaged. However, this apparent drawback is also consistent with high variability reported in cohort studies and clinical cases of infants investigated in neonatal intensive care units.

The most popular model of neonatal HI was adapted from the Levine rat model and was first described by Rice and Vannucci in 1981 [16]. Occlusion of one carotid artery in rodents combined with systemic hypotension is necessary to induce a detectable lesion. Adaptations of this model include occlusion of the left or right carotid, duration and/or degree of oxygen-deprivation exposure to produce mild, moderate or severe injury. This model was reproduced in mice, mostly in P3–P5 pups, but also at a postnatal age ranging from P7 to P12 [17,18,19]. Strain-related brain injury in neonatal P7 HI mice should be taken into account, as some strains (CD1) are particularly susceptible to HI damage, while others (129Sv) are resistant [20]. 

Besides these models, more recent rat pup stroke models were designed to investigate the effects of pure ischemia without hypoxia, and the process of arterial reperfusion injury. These models were performed using Wistar rats on P7 [21], P10 [22] and P14 [23], and Sprague-Dawley rat pups on P7 [24]. All ischemic models in the rat combine transient occlusion of two arteries at the same time, either permanent occlusion of the middle cerebral artery (pMCAo) with transient common carotid artery (CCA) occlusion [25], or blockade of the past ECA (external carotid artery)-ICA (internal carotid artery) bifurcation [24]. Occlusion of both arteries is necessary to create a low cerebral blood flow and a lesion. In contrast, a single pMCAo in the mouse appears sufficient to create an ischemic lesion [19,26].

Ischemia in the P7 rat and the P9 mouse leads to a lesional process, that extents during several weeks [19,21,26,27], with no detectable difference in lesion volume between males and females. Whether the lesional ongoing process reflects continued tissue destruction even in late phase after occlusion or reduced growth potential of the developing brain is yet unclear.

All reported studies investigated both male and female pups. While some authors reported an absence of sex-differences in lesion size and tissue atrophy at short- and long-term, respectively [28,29], a particular study demonstrated that histological damage was sexually dimorphic in specific brain structures [30]. Hemispherical reductions were observed in the ipsilateral hemisphere in adult rats after HI at P7. However, males exhibited these reductions only when the left carotid was ligated, and conversely females exhibited these reductions when the right carotid was ligated [30]. After HI at P3, female rats exhibited larger histological damage when assessed at adulthood [31]. Another study compared residual brain volume using MRI and showed that more severe injury at P10 and P67 in males than in females subjected to HI at P7, with more severe nervous reflex deficits, memory impairment and hemiplegic paralysis [32]. Conversely, HI females were also reported more severely affected in the striatum and white matter [31], whereas others authors reported that males are more severely affected [33,34]. Males P10 mice had worse damage, seizure scores and forelimb asymmetry than females 3 days, but not 1 day, following HI insult [35].

This discrepancy in the extension of damages between males and females might be related to differences in the neonatal models of cerebral injury, the severity of injury, the stage of development at the injury onset, and the time window of assessment. 

## 3. Sexual Dimorphism in Cell Death Pathways

Preclinical studies demonstrated that many signaling cascades are influenced by sex and hormones and activate different cell death pathways in adult rodents subjected to stroke. These sex-dependent cell death pathways were also reported in the developing brain. Increasing data have shown that apoptotic cell death is more pronounced in immature brains than juvenile and adult mature brains [33]. Interestingly, both in vivo and in vitro studies show that the extent of caspase-3 cleavage following brain injury appears to be maximal in the neonatal period and declines with maturation [36,37]. Female cell death is predominantly triggered by the activation of caspase-dependent pathways, whereas ischemia (and/or HI) in males triggers the caspase-independent, poly(ADP-ribose) polymerase (PARP)-dependent cell death pathway [38]. Cleaved caspase-3 was highly detected in female cortical tissues during the first 24 h after ischemia, whereas the detection was low in male cortical tissues [38]. Indeed, females were strongly protected after Q-VD-OPh, a third generation dipeptidyl broad-spectrum caspase inhibitor, as compared to treated males after neonatal stroke in P7 rats [39]. Males are more vulnerable to oxidative stress leading to an important production of reactive oxygen species (ROS) into the mitochondria, large mitochondrial permeability, and maybe opening of the permeability transition pore (mPTP) with a subsequent release of proapoptotic proteins (apoptosis-inducing factor [AIF], cytochrome *c*). In the nucleus, there is an activation of PARP-1 essential to the translocation of mitochondrial AIF into the nucleus to cleave DNA, leading to a caspase-independent cell death [38]. In females release of cytochrome *c* in the cytoplasm activates caspase-3 (leading to cleaved caspase-3), which translocates into the nucleus to cleave PARP-1 and causes caspase-dependent cell death. PARP-1 gene disruption preferentially protects males from perinatal brain injury in P7 (CD-1/sv129 KO) mice as evaluated 10 days after HI [28]. PJ-34, a selective PARP-1 inhibitor, also preferentially protects males from ischemic injury in P9 (C57Bl/6) mice 3 days after injury. A family of proteins, known as inhibitors of apoptosis (IAPs), serves as endogenous inhibitors of cell death, including X-linked IAP (XIAP) recognized to be the most potent by inhibiting caspase-3 cleavage [40]. Since XIAP acts specifically on the caspase-dependent cell death, XIAP may play a role in the selective protection afforded to females.

Autophagy, also known as a non-apoptotic form of programmed cell death [41], is the major mechanism by which cells degrade long-lived proteins and the only known pathway for degrading organelles [42] and may occur with apoptotic cell death in neighboring cells. Sex- and region-specific changes of autophagy in the brain following HI were reported. Females have greater basal autophagy activity than males which may protect cells against injury by increasing cell turn-over [43]. A partial failure to degrade LC3B-II protein (autophagosomal marker which correlates with autophagosome formation) in cortical but not hippocampal lysosomes of females as compared to males following neonatal HI was reported [43]. This blockade of autophagy may commit cells to die by apoptosis, the two processes not excluding each other. In contrast, autophagy is activated in the hippocampus both in males and females following HI [43], response that could be considered as a survival response.

Interestingly, mitochondria seem to be the central point of sexual dimorphism after ischemia and hypoxia-ischemia. Ischemic injury produces different effects on mitochondrial function in males and females at short term that engage distinct cell death and neuroinflammation pathways leading to distinct motor and cognitive deficits in adulthood. Mitophagy is a selective form of autophagy in which damaged or dysfunctional mitochondria are specifically targeted by autophagosomes for lysosomal degradation. Mitochondrial fragmentation occurs following HI in P8 rats both in contralateral and ipsilateral hemispheres to different degrees, in both sexes. However, a sexually dimorphic induction of mitophagy (mitochondrial proteins elimination via mitochondrial-specific autophagy) was described following HI in female, but not in male brain. An accumulation of ubiquitinated mitochondrial protein was observed in male, but not in female HI brains [44].

## 4. Sexual Dimorphism in Oxidative Stress

Mitochondrial dysfunction and oxidative stress were associated with ischemia-reperfusion injury [45] leading to accumulation of ROS and cell death during secondary phase of injury. At the same time, the immature brain is poorly able to handle oxidative stress, with low antioxidant activities that are only half of adult levels. ROS production is related to reoxygenation and reperfusion, with partial recovery of oxidative metabolism, higher level of intracellular calcium and mitochondrial dysfunction [46]. A sexual dimorphism was observed in mitochondrial function. Although respiratory chain activities were inhibited in both sexes with increased depolarization following HI, mitochondrial mass decreased in neonatal female brain but not in males [47] while respiratory chain from is more impaired in the male rat brain [48]. The levels of representative mitochondrial proteins present in the electron transport chain complexes I, II and IV increased substantially one day after HI in female, but not in male brains [49].

Some of the most important sources of ROS are xanthine oxidase products. Allopurinol is a xanthine oxidase inhibitor, one of the main pro-oxidant pathways after HI that inhibits the conversion of hypoxanthine into xanthine and uric acid, thereby limiting the toxic overproduction of ROS. Reduction of perinatal HI brain damage with allopurinol was reported [50], and this reduction appeared to be sex-dependent with females showing decreased markers of apoptosis as compared to males [51]. N-acetylcysteine (NAC) serves as a source of cysteine, a glutathione precursor, and scavenges ROS with its thiol-reducing group, acting directly and indirectly as a potent antioxidant [52]. NAC decreased infarct volumes at 48 h only in female rats when given during hypothermia [53].

Several lines of evidence support the key role of neuronal nitric oxide synthases (nNOS) and endothelial NOS (eNOS) in cerebral blood flow regulation in the acute phase of ischemia and/or HI, and modulation of lesion size after reperfusion-reoxygenation [54]. The selective inhibition of eNOS by L-NIO [55] is associated with a sex-dependent increase in blood flow during ischemia and reperfusion in males but not in females [55], leading to a reduced lesion in females. This increase in blood flow was accompanied by a decrease in nNOS phosphorylation and the production of 3-nitrotyrosine (a marker of oxidative stress) in males. Conversely, selective inhibition of nNOS did not increase blood flow in males and reduced the lesion as compared to treated females [55]. Sex difference in nNOS mRNA was also reported with higher levels in females than males after HI [53]. One study using the NOS inhibitor 2-iminobiotin, reducing endogenous NO production, showed neuroprotection in females but not males after HI, via a NO-independent pathway [56]. Inhaled NO during ischemia was shown to reduce brain injury by selectively dilating arterioles in the penumbra and thereby increasing collateral blood flow [57]. This effect was demonstrated with a sex-dependent effect, protecting males but not females from neonatal HI in the mouse brain [58].

## 5. Sexual Dimorphism and Microglial Activation

Post-ischemic neuro-inflammation could contribute to white matter injury and neurobehavioral disorders described in infants subjected to perinatal brain insult, in particular following stroke or HIE. Neuro-inflammatory is recognized to be a combination of abnormal activation of microglial cells and circulating blood cells that infiltrate brain parenchyma after injury. According to its environment, microglia can adopt several phenotypes (cytotoxic activation, immunomodulatory and repair). Several lines of evidence demonstrate that microglia can play both injurious [59,60] and beneficial [61] role after ischemia.

Very few data are available regarding how microglia number, morphology and activation could differ between males and females. Just before parturition, there are no differences in the number of microglia found in the fetal brain between males and females [62]. However, sex differences in both number and morphology (and gene expression) begin after birth with more microglia in males compared to females at P4 within the parietal cortex, CA1, CA3 and dentate gyrus regions of the hippocampus and the amygdala. At P30 (juvenile) and P60 (early adulthood) female rats have significantly more microglia than males in subregions of the hippocampus as well as the amygdala and parietal cortex [62]. Other studies report a sexual dimorphism in microglia numbers and expression of activation markers in neonatal brains under basal conditions [63,64,65]. In particular, neonatal males had twice as many ameboid microglia as females and a more activated morphological profile [12]. These data strongly suggest that immune cells in the brain play a crucial role in the sexual differentiation of brain and behavior, as well as sex-dependent vulnerability during brain development [12,62,64,65,66,67]. It is unclear whether these sex differences are due to genetic or hormonal causes, but gonadal hormones are known to reduce neuro-inflammation through, as stated above, the response of glial cells to injury including microglial activation.

## 6. Sex Dimorphism and Gonadal Hormones

Cerebral ischemia has sexually dimorphic long-term consequences depending on the developmental stage with possible mechanisms that could be age-dependent [68]. In pre-menopausal women, lower incidence of stroke has been attributed to the protective effects and anti-inflammatory properties of estrogen [69,70]. In early childhood, circulating levels of gonadal hormones are similar in males and in females suggesting that these serum concentrations unlikely mediate the sexually dimorphic phenotype of HI-induced neonatal brain damage during the acute phase [71,72]. Moreover, neonatal HIE leads to an equivalent level of primary brain injury in the male and in female one day after injury [35]. In contrast, secondary neuronal damage and subsequent brain repair could be elicited by an indirect effect of gonadal hormones through the modulation of microglial activation. Indeed, microglial activation and aggregation is a major feature for HIE in human infants [73]. Some data suggest that microglial activation is delayed in neonatal ischemic brains 3 days after the insult. In addition, microglia homeostasis and maturation/activation are not similar in males and in females and exhibit sex-specific profiles under normal and pathological conditions [74].

Several reports have shown that estradiol could mediate anti-inflammatory properties in microglial cells [75,76] with a reduction in the production and secretion of pro-inflammatory cytokines and a downregulation of the transcription of pro-inflammatory genes [77]. Neuroinflammation induced by the excitotoxic insult has been also reported to be a target of estradiol, treatment associated with a reduction in the density of activated microglia [78]. A recent study has reported that gonadal steroids could regulate hypoxia-induced neuro-inflammatory and the microglial phenotype in rat primary microglia in vitro [79]. Favrais et al. have demonstrated that neuroinflammation induces a blockade in the maturation of the oligodendroglial lineage [80]. The attenuation of neuroinflammation through estradiol could therefore enhance white matter maturation and overall brain repair following a perinatal insult.

## 7. Conclusions

Male sex is a well-established epidemiological risk factor for poor neurodevelopmental outcome after perinatal brain injury while the mechanisms responsible for this gender difference are unknown. Growing evidence has identified neuro-inflammation, oxidative stress and cell death pathways as key players in these differences (Figure 1). However, more precise signaling pathways and intimate mechanisms remain unclear. Further studies are urgently needed to investigate the molecular basis of sex-related vulnerability to brain damage and develop new individualized neuroprotective strategies, in the developing brain. As there are few differences in sex hormones during childhood, epigenetic regulation might account for sexual dimorphic phenotypes of neonatal stroke and HI. MicroRNAs (miRs) are important posttranscriptional regulators of gene expression that function by inhibiting the translation of select target genes. MiRs regulate the microglial neuroinflammatory response and miR-targeted therapies were demonstrated to improve clinical outcome following adult cerebral ischemia in pre-clinical studies [81,82,83,84]. Findings of selective miRs involved in the inflammatory responses after acute HI/stroke in newborns boys and girls may further help the clinicians in neonatal intensive care units.
